# Relationships between HDL-C, hs-CRP, with Central Arterial Stiffness in Apparently Healthy People Undergoing a General Health Examination

**DOI:** 10.1371/journal.pone.0081778

**Published:** 2013-12-03

**Authors:** Xi Wang, YingZhen Du, Li Fan, Ping Ye, Ying Yuan, XueChun Lu, Fan Wang, Qiang Zeng

**Affiliations:** 1 Department of Geriatric Cardiology, Chinese PLA General Hospital, Beijing, China; 2 Department of Geriatric Respiratory, Chinese PLA General Hospital, Beijing, China; 3 Second Department of Internal Medicine, Affiliated Hospital of Institute of Aviation Medicine, Air Force, Beijing, China; 4 International Medical Centre, Chinese PLA General Hospital, Beijing, China; University of Catania, Italy

## Abstract

**Background:**

Some cardiovascular risk factors have been confirmed to be positively correlated with arterial stiffness. However, it is unclear whether HDL-C, a well-established anti-risk factor, has an independent association with arterial stiffness. The aim of this study was to evaluate the relationship between HDL-C levels and arterial stiffness and the possible role of high-sensitivity C-reactive protein (hs-CRP) in this potential correlation in apparently healthy adults undergoing a general health examination in China.

**Materials and Methods:**

This was a cross-sectional survey. In total, 15,302 participants (age range, 18–82 years; mean, 43.88±8.44 years) were recruited during routine health status examinations. A questionnaire was used and we measured the body mass index, systolic and diastolic blood pressure, and fasting glucose, and serum lipid, uric acid, hs-CRP, and serum creatinine levels of each participant. Central arterial stiffness was assessed by carotid–femoral pulse wave velocity (cf-PWV).

**Results:**

HDL-C levels decreased as cf-PWV increased. Pearson’s correlation analysis revealed that HDL-C levels were associated with cf-PWV (r=−0.18, P<0.001). hs-CRP levels were positively associated with cf-PWV (r=0.13). After adjustment for all confounders, HDL-C was inversely independently associated with all quartiles of cf-PWV. Furthermore, HDL-C was associated with cf-PWV in different quartiles of hs-CRP, and the correlation coefficients (r) gradually decreased with increasing hs-CRP levels (quartiles 1–4).

**Conclusions:**

HDL-C is inversely independently associated with central arterial stiffness. The anti-inflammatory activity of HDL-C may mediate its relationship with cf-PWV. Further, long-term follow-up studies are needed to evaluate whether high HDL-C levels are protective against central artery stiffening through the anti-inflammatory activity of HDL-C.

## Introduction

Arterial stiffness is an independent predictive factor for all-cause mortality, cardiovascular mortality, fatal and nonfatal coronary events, and fatal strokes in subjects with various levels of cardiovascular risk [1]. Some cardiovascular risk factors such as age, hypertension, and obesity have been confirmed to be positively correlated with arterial stiffness [2,3]. As a well-established anti-risk factor, the cardioprotective effects of HDL cholesterol (HDL-C) have been documented by clinical and experimental research [4,5]. However, it is unclear whether HDL-C has independent protective effects against arterial stiffness. Epidemiological data investigating the relationship between HDL-C and arterial stiffness are limited, and the results are also inconsistent [6]. 

Clinical trials designed to elevate HDL-C levels revealed that with each increment of 1 mg/dL in HDL-C, the risk for complications of coronary heart disease decreased by 2–3% [7]. The beneficial effect of HDL has been largely attributed to its key role in reverse cholesterol transport (RCT) that leads to lipid unloading of the plaque. Accumulating evidence indicates that HDL exerts beneficial effects on cardiovascular pathologies due to its effects on RCT and through pleiotropic effects on vessel wall biology, such as the stimulation of endothelial nitric oxide production and repair, as well as its anti-apoptotic, anti-inflammatory, and anti-thrombotic properties [8]. Some clinical trials revealed the relationship between inflammation and arterial stiffness [9]. Accordingly, we hypothesized that HDL-C is associated with arterial stiffness, at least in part through its anti-inflammatory activity. In the present study, we investigated the correlation between HDL-C and carotid–femoral pulse wave velocity (cf-PWV), a gold standard for evaluating central arterial stiffness, and the possible role of high-sensitivity C-reactive protein (hs-CRP) in this potential correlation in apparently healthy participants undergoing a general health examination in China. 

## Materials and Methods

The study protocol was approved by the Ethics Committee of the Chinese People’s Liberation Army General Hospital (Beijing, China). Each participant provided written informed consent to be included in the study.

### Study population

This cross-sectional survey was designed to establish the relationship between HDL-C and central arterial stiffness through a general health status examination in the health check-up center of our hospital between January 2008 and December 2010. A total of 26,118 adults were recruited. They were ethnically homogeneous (all Han Chinese). All participants were free of malignant tumors, bedridden status, mental disorders, and severe heart and lung failure, and no participant required dialysis. Pregnant females and individuals taking medication known to affect HDL-C metabolism were excluded. In the current study, we also excluded subjects with hypertension (n=9030), diabetes (n=3615), and/or coronary artery disease (CAD) (n=538). Furthermore, we excluded subjects with missing data for essential variables (n=139), resulting in a final sample size of 15,302. Of those excluded due to missing data for essential variables, 43 had missing height and weight information; 21 had missing systolic blood pressure (SBP), diastolic blood pressure (DBP), and heart rate (HR) measurements; and 102 had missing fasting plasma glucose (FPG), serum total cholesterol (TC), triglyceride (TG), low-density lipoprotein cholesterol (LDL-C), HDL-C, hs-CRP, uric acid, and serum creatinine values. 

### Questionnaire and anthropometric measurements

Information about the age, smoking status, history of hypertension, diabetes mellitus, CAD, and medication use of the participants was obtained using standardized self-report questionnaires. This questionnaire was administered using a face-to-face counseling method. The investigation was completed by physicians in the health check-up center of the People’s Liberation Army General Hospital who were trained by the research team. Smoking status was assessed by asking each individual whether he/she was a current smoker.

The physical examination included anthropometric, blood pressure, and heart rate measurements. Height and weight were measured. The body mass index (BMI) was calculated as the weight in kilograms divided by the height in meters squared (kg/m^2^). The blood pressure and HR of the participants were measured in the supine position. The blood pressure measurement was performed using a calibrated desktop sphygmomanometer (Yuyue, Armamentarium Limited Company, Jiangsu, China) after the participants had been in the supine position for ≥5 min, consistent with current recommendations [10]. Blood pressure was measured three times consecutively with ≥1 min between measurements. The mean value of blood pressure was used for the statistical analysis. HR was counted over 60 s.

### Laboratory measurements

Samples of venous blood were collected by venipuncture after an overnight fast. Blood samples (10 mL) were routinely stored at 4°C and delivered to the Department of Biochemistry and the laboratory of the Nephrology Department, People’s Liberation Army General Hospital on the same day. Concentrations of fasting glucose, total cholesterol, triglyceride, HDL-C, LDL-C, hs-CRP, and uric acid were determined using the respective enzymatic assays (Roche Diagnostics GmbH, Mannheim, Germany) on an autoanalyzer (Roche Diagnostics, Indianapolis, IN, USA) in the Department of Biochemistry. Serum creatinine concentrations were measured using an enzymatic assay (Roche Diagnostics GmbH) on a Hitachi 7600 Autoanalyzer (Hitachi, Tokyo, Japan) in the laboratory of the Nephrology Department. All testing was undertaken by well-trained personnel who were blinded to the clinical data. The estimated glomerular filtration rate (eGFR) was calculated using the Chinese modified Modification of Diet in Renal Disease equation [11,12].

### Evaluation of central arterial stiffness

Central arterial stiffness was presented as cf-PWV. Measurements of cf-PWV were performed in a quiet environment in the morning. Subjects were requested to abstain from caffeine, smoking, and alcohol for ≥12 h before the assessment of arterial properties was performed. Subjects were studied in the supine position after resting for 5–10 min. cf-PWV was measured using the Complior Colson device (Createch Industrie, Paris, France), the technical characteristics of which have been described previously [13]. cf-PWV along the artery was measured using two strain-gauge transducers [non-invasively using a TY-306 Fukuda pressure-sensitive transducer (Fukuda Denshi Company, Tokyo, Japan)] fixed transcutaneously over the course of a pair of arteries separated by a known distance. The femoral arteries (on the right side) were used. During preprocessing analyses, the gain of each waveform was adjusted to obtain an equal signal for the two waveforms. During cf-PWV measurements, after pulse waveforms of sufficient quality were recorded, the digitization process was initiated by the operator, and automatic calculation of the time delay between two upstrokes was initiated. The measurement was repeated over 10 cardiac cycles, and the mean value was used for the final analysis. cf-PWV was calculated from the measurements of the pulse transit time and the distance traveled by the pulse between the two recording sites (measured on the surface of the body in meters) according to the following formula: PWV = distance (m)/transit time (s). 

### Statistical analysis

Continuous variables are expressed as the mean and standard deviation (SD). Normality was tested using the Kolmogorov–Smirnov criterion. Skewed variables are expressed as the median value (with an interquartile range). Categorical variables are expressed as numbers and percentages. Baseline characteristics were separated according to the cf-PWV quartiles. cf-PWV levels were classified as follows: quartile 1 (≤7.93 m/s), quartile 2 (7.94–8.78 m/s), quartile 3 (8.79–9.55 m/s), and quartile 4 (≥9.56 m/s). Statistical comparison of the groups was undertaken by one-way ANOVA (continuous variables) or the chi-squared test (categorical variables).

To evaluate the correlation between HDL-C levels and cf-PWV as a continuous variable, Pearson’s correlation analysis for continuous variables or Spearman’s correlation analysis for categorical variables was used in univariate analyses.

In addition, to better understand the correlation between HDL-C levels and different cf-PWV quartiles, logistic regression models were used. Forward stepwise multivariate logistic regression was performed to obtain the odds ratios (ORs) and 95% confidence intervals (CIs) for variables with a probability value ≤0.10, and those with a probability value <0.05 remained in the model after adjustment. Quartile 1 of cf-PWV was used as the reference. Regression models were adjusted for age, gender, and smoking status (model 1), and model 2 was adjusted for model 1 variables plus BMI, SBP, DBP, and HR. Model 3 was adjusted for model 2 variables plus fasting glucose, triglyceride, LDL-C, hs-CRP, eGFR, and uric acid levels. 

To study the role of hs-CRP in the relationship between HDL-C and cf-PWV, the age-adjusted relationship between HDL-C and cf-PWV was investigated for the different hs-CRP quartiles. hs-CRP levels were classified as follows: quartile 1 (≤0.04 mg/dL), quartile 2 (0.05–0.13 mg/dL), quartile 3 (0.14–0.32 mg/dL), and quartile 4 (≥0.33 mg/dL). Pearson’s correlation analysis was used.

All data entry and management activities were undertaken on an Excel spreadsheet, and data were analyzed using SAS statistical software (SAS Institute Incorporated, Cary, NC, USA), version 9.1. A two-sided P-value <0.05 was considered significant. 

## Results

### Characteristics of participants

In total, 15,302 subjects were included in the current analysis. The sample consisted of 9169 males (59.92%) and 6133 females (40.08%). The age range was 18–82 years, and the mean age was 43.88±8.44 years. The study included 5209 (34.0%) subjects who were less than 40 years old. The study also consisted of 9583 (62.6%) subjects between the ages of 40 and 60 years old, whereas only 510 (3.3%) subject were more than 60 years old. There were 5288 smokers (34.6%). 

Table 1 shows the clinical characteristics of the study participants. The participants were divided into four groups based on cf-PWV quartiles. Compared to participants in quartile 1 of cf-PWV, participants in quartile 4 were more likely to be male, older, and smokers, and they had higher values for SBP, total cholesterol, triglyceride, hs-CRP, and uric acid (P<0.05) and lower values for HDL-C (P<0.05). Conversely, the values for DBP, heart rate, BMI, fasting glucose, LDL-C, and eGFR were not significantly different (P>0.05) between quartiles 1 and 4. Furthermore, HDL-C levels declined and hs-CRP levels increased as cf-PWV increased (indicating greater stiffness).

**Table 1:** The clinical characteristics of the study participants.

**Characteristic**	**Overall**	**Quartile 1**	**Quartile 2**	**Quartile 3**	**Quartile 4**
	**(n=15,302)**	**≤7.93**	**7.94–8.78**	**8.79–9.55**	**≥9.56**
		**(n=3848)**	**(n=3841)**	**(n=3817)**	**(n=3796)**
Age (years)	43.88±8.44	42.16±8.27	43.07±8.30	44.13±8.16	46.20±8.50*
<40	5209 (34.0%)	1599 (41.6%)	1419 (36.9%)	1231 (32.3%)	960 (25.3%)
40–60	9583 (62.6%)	2176 (56.5%)	2316 (60.3%)	2474 (64.8%)	2617 (68.9%)
>60	510 (3.3%)	73 (1.9%)	106 (2.8%)	112 (2.9%)	219 (5.8%)
male sex [n (%)]	9169 (59.9%)	1871 (48.6%)	2240 (58.3%)	2491 (65.3%)	2567 (67.6%)*
Current smoking [n (%)]	5288 (34.6%)	1134 (29.5%)	1296 (33.7%)	1400 (36.7%)	1458 (38.4%)**
BMI (kg/m^2^)	24.50±3.21	24.41±3.41	24.54±3.26	24.57±3.12	24.47±3.03
Systolic BP (mm Hg)	112.64±11.34	109.63±11.02	111.55±11.29	113.03±11.05	116.40±10.91*
Diastolic BP (mm Hg)	75.32±8.02	72.77±8.12	74.47±7.91	76.01±7.71	78.07±7.37
Heart rate (min^-1^)	69.06±8.07	68.86±7.53	68.73±7.98	68.97±8.05	69.68±8.65
Total cholesterol (mmol/L)	4.80±0.91	4.68±0.91	4.76±0.90	4.83±0.88	4.92±0.92*
Triglyceride (mmol/L)	1.38 (0.96–2.01)	1.23 (0.85–1.82)	1.32 (0.93–1.96)	1.39 (1.00–2.04)*	1.51 (1.11–2.16)**
LDL cholesterol (mmol/L)	2.59±0.68	2.50±0.68	2.57±0.69	2.65±0.67	2.65±0.69
HDL cholesterol (mmol/L)	1.37±0.36	1.40±0.36	1.37±0.37	1.29±0.36	1.14±0.35*
Fasting glucose (mmol/L)	5.45±0.52	5.44±0.54	5.44±0.53	5.45±0.51	5.46±0.50
Uric acid (mmol/L)	311.68±90.08	301.00±89.46	308.43±91.40*	314.10±89.61*	323.35±88.35**
eGFR (mL/min/1.73 m^2^)	91.66±11.35	92.04±11.29	92.31±11.56	91.52±11.25	90.74±11.23
CRP (mg/dL)	0.19±0.04	0.15±0.07	0.19±0.03	0.21±0.05*	0.21±0.03*
Quartile 1	0.02±0.01	0.02±0.01	0.02±0.01	0.02±0.01	0.02±0.01
Quartile 2	0.07±0.03	0.08±0.02	0.07±0.01	0.69±0.02	0.07±0.02
Quartile 3	0.23±0.02	0.21±0.05	0.24±0.01	0.25±0.04	0.23±0.03
Quartile 4	0.72±0.02	0.68±0.01	0.67±0.07	0.75±0.04	0.78±0.05
cf-PWV (m s^−1^)	8.74±1.29	7.10±0.60	8.40±0.24*	9.13±0.23*	10.37±0.74*

Note: Characteristics are reported as percentages for categorical variables and means (±SD) or medians (with interquartile ranges) for continuous variables. The study participants were divided into four groups based on cf-PWV quartiles (≤7.93, 7.94–8.78, 8.79–9.55, and ≥9.56 m s^−1^). Categorical variables are presented as counts and percentages. The values outside the parentheses are the number of subjects, and the prevalence is presented in parentheses.

The first quartile of cf-PWV was used as the reference.

^*^ P<0.05 vs. Quartile 1.

^**^ P<0.01 vs. Quartile 1.

Abbreviations: eGFR, estimated glomerular filtration rate; BMI, body mass index; BP, blood pressure; HDL, high-density lipoprotein; LDL, low-density lipoprotein; CRP, C-reactive protein; cf-PWV, carotid–femoral pulse wave velocity.

### The association of HDL-C with cf-PWV among the participants

We performed univariate analysis of the relationship between HDL-C and cf-PWV as a continuous variable in all participants. Table 2 shows the result of Pearson’s correlation analyses, which indicated that HDL-C levels were negatively associated with cf-PWV (r=−0.18, P<0.001), whereas hs-CRP levels were positive associated with cf-PWV (r=0.13, P<0.001). In addition, male gender, age, diabetes mellitus, BMI, and triglyceride, LDL-C, hs-CRP, and uric acid levels were associated with cf-PWV.

**Table 2:** Pearson’s correlation between HDL-C and cf-PWV.

	**Univariate**	
**Characteristics**	**r**	**P**
Age (years)	0.19	<0.0001
Male	−0.15	<0.0001
Current smoking	0.07	<0.0001
BMI (kg/m^2^)	0.00	0.8732
Systolic BP (mm Hg)	0.22	<0.0001
Diastolic BP(mm Hg)	0.25	<0.0001
Heart rate (min^−1^)	0.05	<0.0001
Triglyceride (mmol/L)	0.07	<0.0001
LDL cholesterol (mmol/L)	0.08	<0.0001
HDL cholesterol (mmol/L)	−0.18	0.0013
Uric acid (mmol/L)	0.10	<0.0001
Fasting glucose (mmol/L)	0.01	0.0918
CRP	0.13	<0.0001
eGFR (mL/min/1.73 m^2^)	−0.05	<0.0001

Abbreviations: BMI, body mass index; BP, blood pressure; HDL, high-density lipoprotein; LDL, low-density lipoprotein; CRP, C-reactive protein; eGFR, estimated glomerular filtration rate.

The relationship between HDL-C and different quartiles of cf-PWV among the participants is shown in Table 3. A stepwise logistic regression model was created, and quartile 1 of eGFR was used as the reference. The results illustrated that after adjustment for all confounders including age, gender, smoking status, BMI, SBP, DBP, HR, triglyceride, LDL-C, fasting glucose, hs-CRP, eGFR, and uric acid (model 4), HDL-C was inversely independently associated with all quartiles of cf-PWV. Furthermore, the OR decreased gradually as cf-PWV increased (quartiles 2–4) (indicating greater stiffness).

**Table 3:** Correlation between HDL-C and cf-PWV in all participants.

	**Quartile 1**	**Quartile 2**	**Quartile 3**	**Quartile 4**
	**≤7.93**	**7.94–8.78**	**8.79–9.55**	**≥9.56**
**Model 1**				
Odds ratio	1	1.02	0.94	0.87
95% CI	Reference	0.87–1.19	0.82–1.08	0.75–1.01
P		0.843	0.391	0.060
**Model 2**				
Odds ratio	1	0.95	0.89	0.79
95% CI	Reference	0.82–1.09	0.66–1.18	0.68–0.92
P		0.433	0.088	**0.003**
**Model 3**				
Odds ratio	1	0.89	0.80	0.64
95% CI	Reference	0.76–0.98	0.69–0.93	0.47–0.84
P		**0.033**	**0.006**	**<0.001**

Note:Model 1: Adjusted for age, gender, and smoking status.

Model 2.

Adjusted for model 1 variables plus body mass index, systolic blood pressure, diastolic blood pressure, and heart rate.

Model 3.

Adjusted for model 2 variables plus fasting glucose, uric acid, total cholesterol, triglyceride, low-density lipoprotein, estimated glomerular filtration rate, and C-reactive protein.

### The possible role of hs-CRP in the relationship between HDL-C and cf-PWV

**Figure 1:**
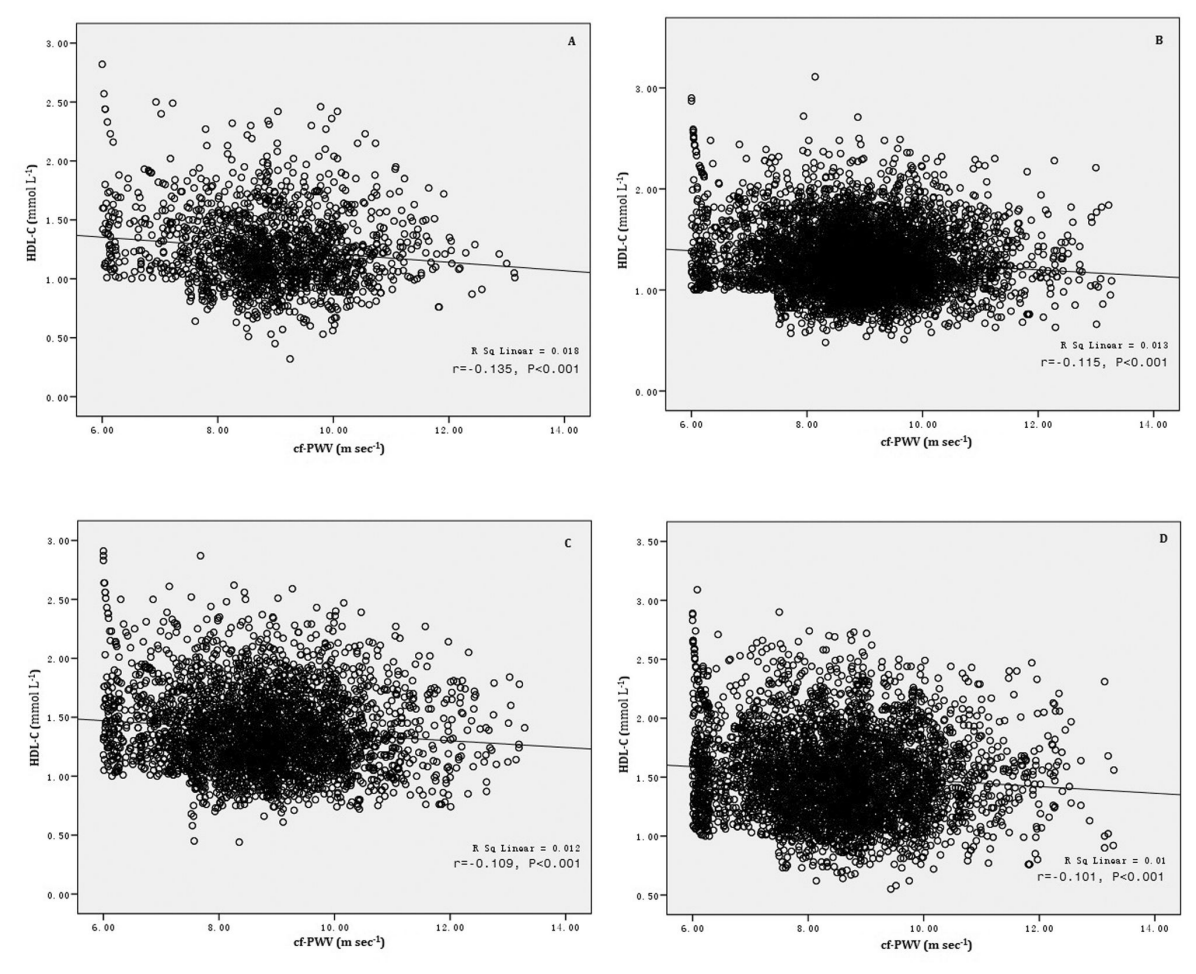
Relationship between HDL-C and cf-PWV in different quartiles of hs-CRP. Pearson’s correlation analysis was used. HDL-C was negatively correlated with Cf-PWV in all quartile of hs-CRP (A to D). The correlation coefficients gradually decreased as hs-CRP increased (quartiles 1–4) (r= −0.135, −0.115, −0.109, and −0.101, respectively, P<0.001). A: Quartile 1 of hs-CRP (≤0.04 mg/dL); B: Quartile 2 of hs-CRP (0.05–0.13 mg/dL); C: Quartile 3 of hs-CRP (0.14–0.32 mg/dL); D: Quartile 4 of hs-CRP (≥0.33 mg/dL). Abbreviations: cf-PWV, carotid–femoral pulse wave velocity; HDL-C, high-density lipoprotein cholesterol; hs-CRP, high-sensitivity C-reactive protein. X-axis: the value of cf-PWV (m s^−1^); Y-axis: the value of HDL-C (mmol/L); r, Pearson’s correlation coefficient.

The possible role of hs-CRP in the relationship between HDL-C and cf-PWV is illustrated in Figure 1 (A-D). Pearson’s correlation analysis for HDL-C and cf-PWV was used for all quartiles of hs-CRP. The results indicated that HDL-C was associated with cf-PWV for all quartiles of hs-CRP (P<0.001). Furthermore, the correlation coefficients (r) gradually decreased as hs-CRP increased (quartiles 1–4). 

## Discussion

In this study, we investigated the correlation between HDL-C, inflammation and central arterial stiffness in apparently healthy participants undergoing a general health examination. The major findings of this study are that even after adjusting for other confounding factors (model 4), HDL-C is inversely and independently associated with cf-PWV, and the OR decreased gradually as cf-PWV increased (quartiles 2–4). Furthermore, the correlation between HDL-C and cf-PWV is attenuated at higher hs-CRP levels. Therefore, these results reveal an independent relationship between HDL-C and central artery stiffness. Moreover, this association is potentially related to the anti-inflammatory effect of HDL-C.

The relationship between serum lipids and central arterial stiffness is rarely investigated because serum lipids are always considered to have a critical role in atherosclerosis. Previous studies also revealed a relationship between total cholesterol or LDL-C and central arterial stiffness [14,15]. However, a systematic review published in *Hypertension* reported that only approximately 10% of studies on the correlation between serum lipids and arterial stiffness suggested a positive association, and most studies failed to identify such a correlation [16]. The most important limitation was that most studies focused on the association between lipids and SBP, DBP, or PP, and data concerning PWV (the current gold standard measure of arterial stiffness) were lacking [17,18]. Meanwhile, epidemiological or clinical data concerning the relationship between HDL-C and central arterial stiffness is rare, and the results are inconsistent due to the use of differing methods to evaluate arterial stiffness. Recently, the cardiovascular protective effect of HDL had been studied, but the results were inconsistent. Wang F et al [13] investigated the relationship between serum lipids and pulse wave velocity (PWV) in community-dwelling individuals in Beijing, China. The results showed HDL-C was independently inversely associated with aortic stiffness and peripheral arterial stiffness. This is the first study to report the relationship between serum lipid and arterial stiffness in Chinese population. Then, Zhao WW et al [19] reported that after multiple adjustments, participants in the highest quartile of HDL-C had an odds ratio of 0.442 (95% CI 0.268-0.729) for developing high arterial stiffness compared with participants in the lowest quartile and HDL-C was related with arterial stiffness. However, the result of another investigation performed in large-scale Chinese population were carry out by Weng C et al [20] revealed the differently completely conclusion. The report showed that HDL-C was not correlated with baPWV in different age and gender groups and of the MetS components, elevated BP was the strongest predictor of brachial-ankle PWV. This results were confirmed by another study performed by the same research team [21]. In the present study of 15,302 apparently healthy participants, we investigated the correlation between HDL-C, inflammation and cf-PWV as a measure of central arterial stiffness. Our results documented that HDL-C was independently inversely associated with central arterial stiffness. Moreover, the possible role of inflammation was investigated in the relationship between HDL-C and central arterial stiffness for the first time compare with the previous studies. The results of the present study would provide more objective and valuable evidences. 

There are several possible mechanisms which HDL-C protects against arterial stiffening. First, the RCT activity of HDL-C is the major mechanism of anti-atherosclerosis, which may be in part to improve arterial stiffness. Second, HDL-C has several direct, non-atheromatous effects on the arterial wall that may improve arterial stiffness [22]. Recently, studies revealed that HDL exerts pleiotropic effects on vessel wall biology, such as the stimulation of endothelial nitric oxide production and repair, as well as anti-apoptotic, anti-inflammatory and anti-thrombotic effects [23-25]. Moreover, inflammation is associated with arterial stiffness, as confirmed by several clinical and experimental studies. Yasmin et al [26] demonstrated a positive relationship between serum matrix metallopeptidase 9 levels and aortic PWV in a large cohort of apparently healthy subjects. Twenty-year follow-up from the Caerphilly Prospective Study confirmed a significant positive relationship between aortic PWV and CRP. Thus, HDL-C and arterial stiffness have a pathophysiological correlation. In the present study, the results indicated that HDL-C was associated with cf-PWV in different quartiles of hs-CRP (P<0.001), and the correlation was gradually attenuated with increasing hs-CRP levels (quartiles 1–4). The results suggested that the anti-inflammatory effect of HDL-C may mediate the relationship between HDL-C and cf-PWV. 

Compare with the previous studies, the highlight of our study was that the role of inflammation was investigated in the relationship between HDL-C and central arterial stiffness. Other publications just studied the relationship between HDL-C and PWV. In our investigation we explored the effect of inflammation. Just like the facts mentioned above, the pleiotropy of HDL maybe have the important effects on arterial stiffness, meanwhile, the reverse cholesterol transport of HDL is responsibility for atherosclerosis. They are different but related concepts and our study provide some significant epidemiological value for deeply researching. 

There are several limitations in the present study. First, because of the cross-sectional design and its inherent limitations, the present study cannot determine causal relationships between the associations. Accordingly, our observations need confirmation in longitudinal and interventional studies. Second, this is a population undergoing a general health examination in the health check-up center of our hospital. The population may have good comprehension and more health requirements, and it may not represent the general population. Third, the study consisted of a small number of subjects older than 60 years and a large number of middle-aged adults, which may have affected the results. Fourth, although the results were adjusted for multiple covariates that may be associated with cf-PWV or altered vascular properties, the possibility of residual confounds remains.

## Conclusions

In conclusion, we observed cross-sectional associations between HDL-C levels and central artery stiffness, and the anti-inflammatory activity of HDL-C may mediate the relationship between HDL-C and cf-PWV. However, long-term follow-up studies are needed to evaluate whether high HDL-C levels have protective effects against central artery stiffening through the anti-inflammatory activity of HDL-C. 
